# X-ray-generated heralded macroscopical quantum entanglement of two nuclear ensembles

**DOI:** 10.1038/srep33361

**Published:** 2016-09-19

**Authors:** Wen-Te Liao, Christoph H. Keitel, Adriana Pálffy

**Affiliations:** 1 Max-Planck-Institut für Kernphysik, Saupfercheckweg 1, D-69117, Heidelberg, Germany; 2 Department of Physics, National Central University, 32001 Taoyuan City, Taiwan

## Abstract

Heralded entanglement between macroscopical samples is an important resource for present quantum technology protocols, allowing quantum communication over large distances. In such protocols, optical photons are typically used as information and entanglement carriers between macroscopic quantum memories placed in remote locations. Here we investigate theoretically a new implementation which employs more robust x-ray quanta to generate heralded entanglement between two crystal-hosted macroscopical nuclear ensembles. Mössbauer nuclei in the two crystals interact collectively with an x-ray spontaneous parametric down conversion photon that generates heralded macroscopical entanglement with coherence times of approximately 100 ns at room temperature. The quantum phase between the entangled crystals can be conveniently manipulated by magnetic field rotations at the samples. The inherent long nuclear coherence times allow also for mechanical manipulations of the samples, for instance to check the stability of entanglement in the x-ray setup. Our results pave the way for first quantum communication protocols that use x-ray qubits.

As a purely quantum mechanical property, quantum entanglement has been demonstrated and is nowadays routinely realized with photons, typically in the long wavelength, optical regime, or with quantum particles such as electrons, ions or atoms^1^,^2^,^3^,^4^,^5^. However, it has been shown that also large, macroscopical collections of the latter may experience entanglement. While from a fundamental point of view this is interesting for the study of the boundary between the quantum realm and the classical world^6^, practically it may be of paramount importance for quantum communication applications. The ability to create entanglement between quantum memories in a heralded manner^5^ can be advantageous for quantum communication developments, for instance for quantum repeaters^1^ and quantum networks^7^. The search for scalable quantum repeaters has come up with solid-state resources, which require entanglement between quantum memories hosted in spatially separated macroscopical crystals^5^. So far, such macroscopical entanglement has been typically limited in either time duration, working temperature or sample size/number of atoms involved, as shown in Table 1 that lists some key achievements. As generic feature, these experiments make use of optical photons as entanglement carriers^2^,^3^,^4^,^5^.

The commissioning of the first x-ray free electron lasers (XFEL)^8^,^9^ and recent developments in x-ray optics^10^,^11^,^12^,^13^ and single x-ray quanta manipulation^14^,^15^,^16^ open new perspectives for quantum information and quantum communication protocols using x-ray qubits. The advantages of higher frequency photons would be better focusing, deeper penetration power, robustness and improved detection^15^. The latter two are in particular pertinent for quantum technology applications, while the improved focusability might help shrinking future photonic devices^14^. X-ray quantum optics^17^ promises so far in theory exciting applications in metrology^18^,^19^ and information technology^15^,^20^, as well as for generation of photon entanglement in the keV regime using nuclear rather than atomic transitions^21^. Due to their more suitable transition energies, nuclei arise as natural candidates for x-ray quantum optics, especially Mössbauer nuclei which allow for a collective, delocalized excitation throughout macroscopic nuclear ensembles.

Here we investigate a scenario to create and manipulate heralded entanglement between two macroscopic solid objects, i.e., crystals containing Mössbauer nuclei, using x-rays. An implementation in the x-ray regime and using for the first time nuclear systems instead of atoms would demonstrate the universality of quantum optics for a new energy regime and degree of complexity. From the practical point of view, such a setup presents several advantages compared to existing realizations, which are (i) room temperature handling, (ii) long coherence times, and (iii) solid-state macroscopical samples with a large number of constituents, particularly appealing for decoherence studies at the borderline between the classical and quantum worlds, (iv) comparing with other setups, we put forward the first scheme to directly manipulate macroscopic entangled objects while other proposals or experiments rather aim at manipulating the single photon that is used to entangle the macroscopic objects. This may provide a stronger evidence of nonlocality of macroscopic entanglement.

An x-ray parametric down conversion (XPDC) process x-ray → x-ray + extreme ultraviolet (X → X + EUV)^22^,^23^ in a diamond crystal provides an x-ray quantum that impinges on an x-ray interferometer^24^ as shown in Fig. 1. The detection of the EUV idler photon at the detector A heralds the presence of the x-ray photon in the setup. In a first step, a 50/50 beam splitter^12^ BS 1 transfers the signal photon into a two-path entanglement state 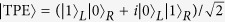, where states 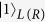 and 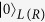 refer to the one-photon Fock state and the vacuum state at the left (right) path, respectively. This single-x-ray entanglement state subsequently reaches two crystals containing ^57^Fe nuclei and may drive the magnetic dipole transition from the nuclear ground to the first excited state at 14.4 keV. With recoilless absorption and reemission of the x-ray photon due to the Mössbauer effect, the nuclear excitation is coherently spread over the entire nuclear ensemble, and remains delocalized. This nuclear exciton state 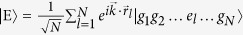 comprises the 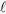
th nucleus at position 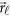 excited by the incident x-ray with the wave number 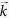, whereas all other (*N* − 1) nuclei remain in their ground state^14^,^25^. As a result of quantum interference between the emission from each crystal site, a directional remission of a single photon along the incident 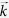 follows the decay of state 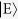. Also known under the name of single-photon superradiance^26^, this coherent, cooperative decay is strongly enhanced compared to the spontaneous decay channel^25^ and is routinely observed in nuclear forward scattering experiments with nuclear solid-state targets typically few *μ*m thick and few mm in diameter^25^. The specific speed-up decay characteristics are determined mostly by the optical thickness of the sample. We note here that since at most only one resonant photon is present in the system at any given time, the superradiance experienced by the Mössbauer nuclei is different from traditional atomic superradiance which involves a strong excitation of the sample.

Due to the incident two-path entanglement state, the resonant scattering on the two remote crystal samples labeled by *L* and *R* leads to the formation of an entangled state 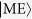 between two distant parties


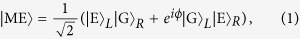


where 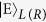 and 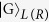 stand for the *L*(*R*) ensemble being in the excited state 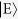 and in the ground state, respectively, and *ϕ* is the relative phase between two components. As additional control parameter, each nuclear sample is under the action of a hyperfine magnetic field, denoted by B_L_ and B_R_, as illustrated in Fig. 1a. The nuclear response is recombined by a second beam splitter BS 2 and monitored by two detectors B and C.

The key for the setup is the arrangement of x-ray and EUV detectors such that without interacting with the nuclear sample crystals, either both detectors A and B, or both detectors A and C simultaneously register the two XPDC photons. Each successful creation of the entanglement state 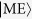 in Eq. (1) is heralded by the click at detector A while no photon is registered at detectors B, C (registering the coherent decay of the nuclear exciton) or any other detectors monitoring the 4*π* emission angle (for photon loss or incoherent, spontaneous decay of the nuclear exciton). Modern x-ray detectors are few up to 10 centimeters in size and much larger than the nuclear samples, thus facilitating a wide solid-angle monitoring. The missing count of an x-ray signal photon is attributed to the absorption by the two remote crystals. The absorbed and rescattered signal photon reaches the detectors B or C and is recorded only later, with a time delay given by the nuclear excited state lifetime. We recall that the latter will be influenced by superradiant decay channels in the system. By choosing a moderate optical thickness 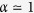, the x-ray emission is predominantly occurring in the forward direction, facilitating detection, while the speed-up of the decay remains relatively small, leading to the signal delay of few tens up to 100 ns. In order to minimize the effects of false non-detection events, which are unavoidable despite high detection efficiency, a valid data record will require the time-delayed coincidence of two detectors, either detectors A and B, or detectors A and C. Thus, false non-detection events will not jeopardize the fidelity of the prepared state, but rather just reduce the rate with which heralded entanglement can be recorded.

For a detectable production rate of the heralded macroscopic entanglement, the key requirement is that the XPDC source produces down-converted x-ray signal photons which are broadband relatively to the nuclear resonance width and cover the hyperfine-split nuclear absorption lines. Over this narrow range, the nuclear resonance absorption exceeds by orders of magnitude the atomic background processes^25^. An estimate of the flux of generated x-ray parametric down conversion photons by an incoming XFEL pulse (see Methods) gives 3 × 10^6^ signal photons/s with a 1 eV bandwidth. This corresponds to a 0.1 Hz production rate of heralded macroscopic entanglement. We expect that the latter rate can be further increased by one or two orders of magnitude, i.e., *R*_*E*_ ~ 10 Hz, by a tighter focusing on a diamond crystal which would enhance the nonlinear efficiency of XPDC. We also note that the signal photon rate is low enough to allow sufficient potential recording time (several hundreds ns) between single shots. Further attention is required for avoiding losses by air absorption of the heralding EUV photon^22^ and also for the mechanical alignment of the setup, with XPDC source, beam splitters and mirrors all having angular acceptances of *μ*rad^11^,^22^,^24^.

To verify the entanglement between the two nuclear sample crystals, we invoke the method of quantum state tomography^3^,^5^,^27^ to determine the density matrix 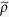 of 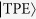 of the coherently re-emitted single x-ray photon from two targets (see Methods). The key quantity to be determined experimentally is the visibility *V* of the interference fringe at detectors B and C. The remitted single photon is allowed to interfere with itself on beam splitter BS 2 for different phase shifts while measuring the interference fringe. Typically, an additional Si phase shifter or a vibrating crystal are used to mechanically vary the phase between the two arms in an interferometer^24^ for the determination of *V*. Here we propose a novel magnetic, non-mechanical solution for phase modulation that directly and locally controls the nuclear dynamics in each ensemble and can provide an indication of entanglement between the two remote parties. The phase modulation can be achieved via a fast rotation of the hyperfine magnetic field at one of the crystals. We note that the magnetic field rotations provide direct control over the entangled macroscopic ensembles rather than over the scattered photons^3^,^4^. In principle, this renders possible new decoherence tests by superimposing mechanical movement in parallel to monitoring the effects of the magnetic phase modulation. More practical aspects related to the mechanical stability requirements of the setup will be discussed in the following.

Due to the hyperfine magnetic field, each ^57^Fe 14.4 keV nuclear transition is split into a sextet (Fig. 1b). The typical bandwidth of the down-converted photons of approx. 1 eV^22^ is much broader than the linewidth of the interacting nuclear transition such that the scattering photon can drive any of the six transitions between the hyperfine ground state and excited state levels. The setup geometry (Fig. 1a) is chosen such that linearly polarized x-ray photons will drive simultaneously the two Δ*m* = 0 transitions, with Zeeman energy shifts ±*ħ*Δ_B_. The quantum coherences in the two crystals (see Methods) are simultaneously driven by the down-converted signal photon and rotate in time as shown by Fig. 1(c,d). Due to the Zeeman shifts ±*ħ*Δ_B_, the two pairs of coherences accumulate a phase 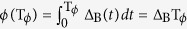 for a constant Δ_B_. If just one of the magnetic fields, for instance B_R_ is inverted at *t* = T_*ϕ*_, the right mode turns into cos(*ϕ* − Δ_B_*t*), whereas the left wavepacket is still proportional to cos(*ϕ* + Δ_B_*t*), leading to a phase shift between the two samples. We thus can magnetically control, without need of a mechanical solution, the quantum phase between the two spatially separated entangled nuclear crystals. Rapid manipulations of the hyperfine magnetic field in iron samples have been demonstrated with antiferromagnetic ^57^FeBO_3_ crystals^28^.

The resulting interference fringe can be analyzed by investigating the output intensities at the two detectors B and C, 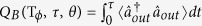 and 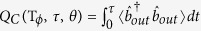, respectively. Here, *τ* is the photon counting time after T_ϕ_, *θ* is the phase shift experienced by the x-ray photon when transmitted by the beam splitters BS 1 and BS 2 and 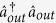 and 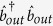 are the photon number operators for the two output fields at detectors B and C, respectively. The output fields can be obtained by considering the action of the beam splitter BS 1, nuclear scattering in samples *L* and *R*, mirror and beam splitter BS 1, respectively, on the incident XPDC field. In matrix form, this can be written as^29^


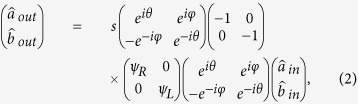


where 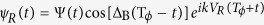 and 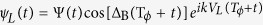 and *θ* and *φ* are the phase shift of the transmitted and reflected x-rays, respectively, relative to the incident x-rays. We assume in Eq. (2) that reflectivity = transmittance = *s*. As the field 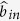 is in the vacuum state, the number operators 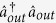 or 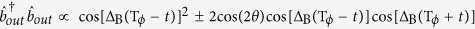

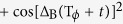
. Numerical results for the interference fringes *Q*_*B*_ and *Q*_*C*_ are presented in Fig. 1e for optical thickness *α* = 1, natural decay rate Γ = 1/141 GHz for ^57^Fe and Δ_*B*_ = 30Γ. The coherence time of the entanglement between two crystals is approx. 60 ns in Fig. 1e, corresponding to the chosen optical thickness value. The lifetime of the entanglement state can be prolonged up to the natural mean lifetime of the ^57^Fe excited state of 141 ns by either rotating^28^ the hyperfine magnetic field as has been demonstrated experimentally in ^57^FeBO_3_ crystals^28^ or by switching it off^14^ to obtain a quantum memory. The fidelity under the action of the quantum memory could be defined as 
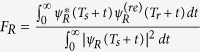
 and 
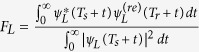
. Both are the measure of the similarity between the retrieved wavepacket 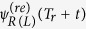 and the wavepacket *ψ*_*R*(*L*)_(*T*_*s*_ + *t*) to be stored, where *T*_*s*_ is the instant of storage, and *T*_*r*_ is the instant of retrieval. Since storage via magnetic field rotation^28^ does not freeze the magnetic phase evolution, we expect that switching off the magnetic field^14^ to have the hyperfine splitting completely vanish may prepare the required state with higher fidelity. We note that 141 ns would be the longest coherence time achieved for macroscopic entanglement of solid-state samples^4^,^5^, see Table 1.

The degree of entanglement may be influenced by the performance of the beam splitters BS 1 and BS 2. Considering imperfect beam splitters, we may write 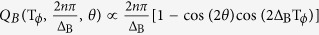 and 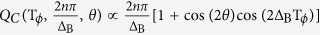 for a *α* < 1 and an integer value *n*. The visibility fringe becomes in this case 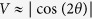, showing that the maximum entanglement occurs at *θ* = *nπ*/2. A further dynamical decoherence mechanism may come into play with vibrations or displacements of the target. In order to simulate this effect theoretically and find out the tolerance vibration of the system, we envisage that the two entangled targets experience movement with random velocities *u* along the direction of photon propagation. Displacements in the plane orthogonal to the photon propagation would lead to misalignments in the interferometer setup and failure to recover the photon at detectors B and C. Let us assume that *u*_*R*_(*t*) and *u*_*L*_(*t*) are random numbers chosen within a certain range for each time instant *t*. Figure 2 demonstrates numerical results with random velocities in the ranges of **a** (−0.1, 0.1) mm/s, **b** (−0.2, 0.2) mm/s and **c** (−0.4, 0.4) mm/s showing that the interference fringes gradually become blurred when extending the maximum vibration speed to *ku*_*R*_ ~ Δ_B_ or *ku*_*L*_ ~ Δ_B_. These values are much larger than the typical vibration fluctuation of a stabilized x-ray interferometer^24^, confirming that macroscopical entanglement can be generated and sustained with the proposed setup.

Mechanical vibrations of the samples act as classical dephasing to the entanglement setup. In particular, random classical dephasing can be used to mimick quantum decoherence^6^. Thus, an experimental observation of the behaviour shown in Fig. 2 may shed light on the nature of quantum decoherence occurring in the entanglement of two crystals with Mössbauer nuclei. This is the unique feature of our setup due to the long nuclear decoherence times, the solid-state nature of the samples and finally to the magnetic-field phase control, which does not require any mechanical manipulation for entanglement checks in the first place. The theoretical treatment of specific decoherence models goes however beyond the scope of this paper.

In conclusion, we have demonstrated a scheme that employs x-ray quanta in a new parameter regime to create macroscopical entanglement between two crystals hosting Mössbauer nuclei. The use of stable and well isolated nuclear systems allows longer coherence times together with room temperature handling. Entanglement relies on a delocalized nuclear excitation which can be spread over a large number of nuclei, for typical sample and focus parameters of up to approx. 10^14^. Quantum state tomography in conjunction with a novel magnetic-phase control technique can be employed to characterize the entanglement state. The entangled crystals can then be subjected to decoherence tests involving mechanical movement. We expect that heralded entanglement using x-rays and nuclear transitions can thus open a new research avenue for both applied ideas related to quantum technology as well as more foundational studies of the boundary between the quantum and classical worlds.

## Methods

**Figure 1:**
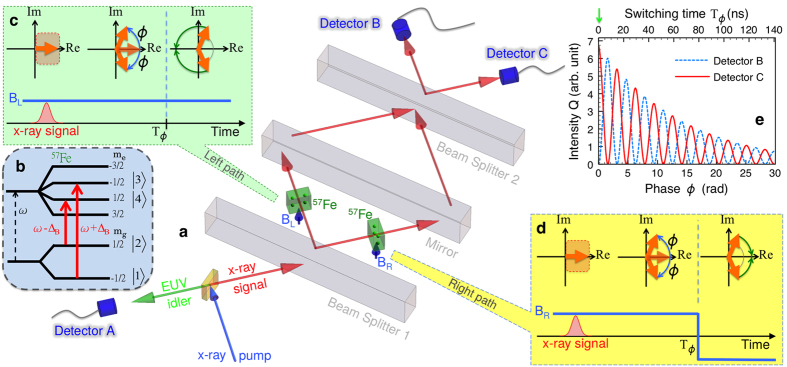
Sketch of the creation of macroscopic entanglement. (**a**) A combination of x-ray interferometry with NFS and an XPDC setup. X → X + EUV down-conversion in an antiparallel geometry occurs within a diamond crystal (yellow cuboid). Subsequently, a converted single x-ray signal photon enters an x-ray interferometer while a converted EUV idler photon reaches detector A producing a click. Beam splitter BS 1 transfers the signal photon into a two-path entanglement state. The photon is then coherently scattered off the two ^57^Fe crystals (green slabs). The nuclear transitions in the latter experience hyperfine splitting under the action of the applied magnetic fields B_L_ and B_R_ (blue short arrows). As the single photon is absorbed and shared by the two distant nuclear crystals, the latter are entangled in the state |ME〉 (1). The re-emitted signal photon from the nuclear crystals is in turn reflected by the mirror, recombined at beam splitter BS 2 and registered by either detector B or C. (**b**) ^57^Fe nuclear level structure. A linear polarized x-ray signal photon drives two Δ*m* = 0 transitions (red solid arrows) with Zeeman-shifted frequencies *ω* ± Δ_*B*_. The black dashed arrow depicts the unshifted transition frequency *ω*. (**c**,**d)** Dynamics of the coherence terms (rotating orange thick arrows) on the left and right arms of the interferometer induced by the time-dependent magnetic fields B_L_ and B_R_ (blue solid lines), respectively. B_R_ is inverted at T_*ϕ*_. (**e**) Interference pattern Q at detectors B and C as a function of the magnetic phase *ϕ*(T_*ϕ*_) and magnetic switching time T_*ϕ*_. The light green downward arrow indicates the moment a click at detector A starts the chronometer for T_*ϕ*_.

For the calculation of the nuclear response we use the well-known Maxwell-Bloch equations^29^: 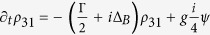, 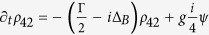 and 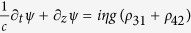. Here, *ρ*_*mn*_ is the coherence between states |*m*〉 and |*n*〉, with {*m, n*} ∈ {1, 2, 3, 4} as depicted in Fig. 1b, 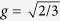 the Clebsch-Gordan coefficient, *c* the speed of light, 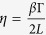 and *L* the crystal thickness. With the boundary condition *ψ*(*t*, 0) = *δ*(*t*) for a broadband incident x-ray, the coherently scattered x-ray wavepacket off the nuclear crystals reads^25^ 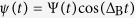, and 
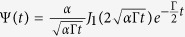
. Here, *J*_1_ is the first order Bessel function of the first kind due to multiple scattering events in the sample, *α* = *g*^2^*β*/2 the effective resonant thickness and Γ the spontaneous decay rate of the nuclear excited state. Furthermore, the trigonometric oscillation is caused by the quantum beat of the two split nuclear transitions, and the exponential decay term describes the incoherent spontaneous decay of the excited states.

Entanglement realization can be checked by means of quantum state tomography. In the photon-number basis the density matrix of the coherently re-emitted single x-ray photon 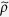 reads^3^,^27^


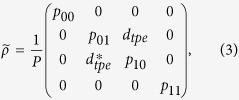


where *p*_*ij*_ is the probability of detecting *i* photons from the left crystal and *j* photons from the right one. Furthermore, *d*_*tpe*_ is the coherence between the two components of |TPE〉 and 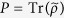. The concurrence 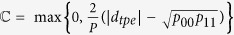 from a measured 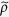 then quantifies a lower bound for entanglement such that 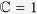 for maximal entanglement and 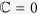 for a pure quantum state^3^,^27^. With the approximation *p*_00_ ≈ 1 − (*p*_01_ + *p*_10_ + *p*_11_), the diagonal terms in Eq. (3) can be determined experimentally by conditional measurements that distinguish between photons scattered by the *L* or *R* samples, e.g., by removing the second beam splitter BS 2. What concerns the coherence term *d*_*tpe*_, it has been shown that this can be approximated as^3^,^27^ *V*(*p*_01_ + *p*_10_)/2, where *V* is the visibility of the interference fringe at detectors B and C.

For an experimental implementation, we now estimate the possible production rate of heralded macroscopic entanglement. With a nuclear resonance cross section of *σ* = 2.5 Mbarn for the 14.4 keV transition of ^57^Fe, already a sample of 20 *μ*m thickness is likely to absorb all incoming resonant photons. Assuming 100% detection efficiency^15^, the flux *R*_*E*_ of produced resonant photons equals the rate of heralded entanglement creation. The flux can be estimated as *R*_*E*_ = *ξ*_*s*_Δ*E*_*n*_/Δ*E*_*s*_, where Δ*E*_*n*_ = 4.66 neV is the linewidth of the considered ^57^Fe nuclear transition, and Δ*E*_*s*_ = 1 eV and *ξ*_*s*_ are the bandwidth and the flux, respectively, of the XPDC signal photons^22^. According to ref. 22, 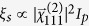, where *I*_*p*_ is the photon density of the pump field, and 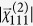 the 111 Fourier coefficient of the second order nonlinear susceptibility for a diamond (111) crystal^30^. By introducing *ω*_*p*_ = *ω*_*s*_ + *ω*_*i*_ 22, we obtain the susceptibility


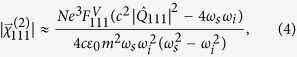


where *ω*_*p*_, *ω*_*s*_, and *ω*_*i*_ are the angular frequencies of pump, signal and idler photons, respectively, *N* is the number density of unit cells, 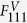 the linear structure factor of bound electrons^22^,^30^ and 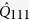 the 111 reciprocal lattice vector of a diamond crystal. Further parameters in Eq. (4) are *m* the electron mass, *e* the electron charge and *ε*_0_ the vacuum permittivity. Given *ℏω*_*s *_= 14.4 keV and *ℏω*_*i*_ = 100 eV, 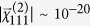 C/N, having the same order of magnitude as for the case of *ℏω*_*s*_ = 10.9 keV reported in ref. 22. Since for the latter, SR pulses were used as pump field, the pump photon density can be enhanced by considering an XFEL pulse. Considering a train of XFEL pulses with 10^12^ photons/pulse and repetition rate^8^ *f* = 2.7 × 10^4^, on a spot size^22^ of 5000 *μ*m^2^, by simple scaling, we then obtain *ξ*_*s*_ = 2.9 × 10^6^ signal photons/s.

## Additional Information

**How to cite this article**: Liao, W.-T. *et al*. X-ray-generated heralded macroscopical quantum entanglement of two nuclear ensembles. *Sci. Rep.* **6**, 33361; doi: 10.1038/srep33361 (2016).

## Figures and Tables

**Figure 2:**
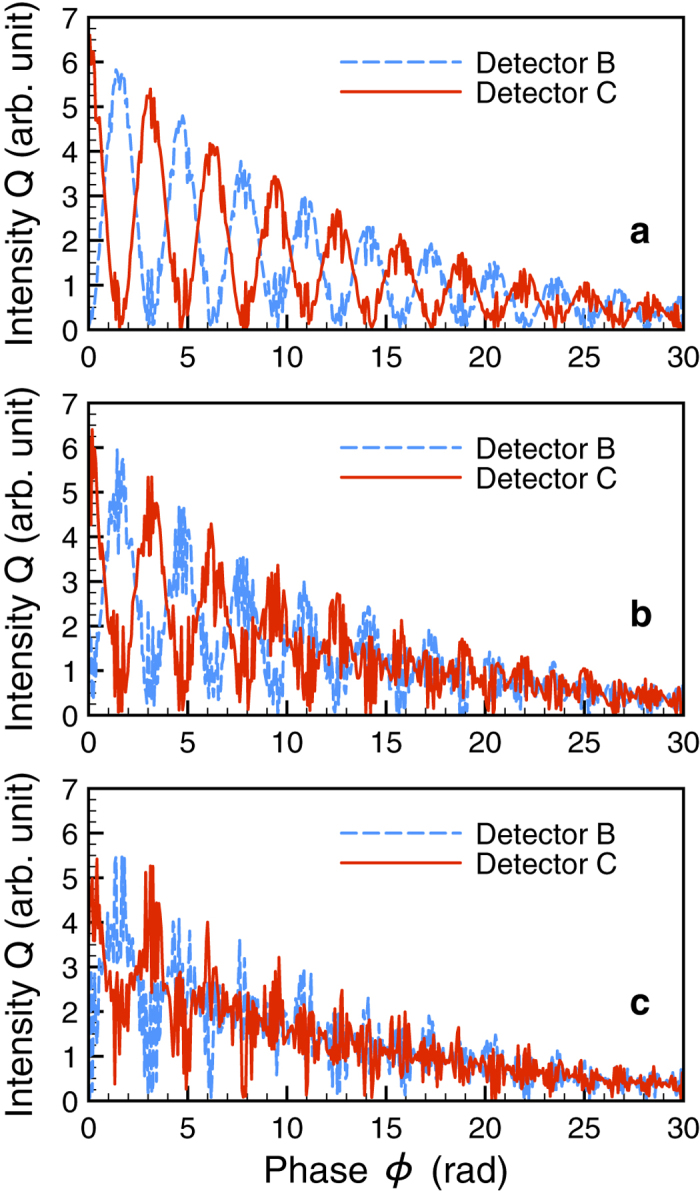
Decoherence caused by the vibration of entangled targets. The corresponding amplitudes of random velocities *u*_*R*_ and *u*_*L*_ are (**a)** ±0.1 mm/s, (**b**) ±0.2 mm/s and (**c**) ±0.4 mm/s. The hyperfine splitting is Δ_B_ = 30Γ.

**Table 1:** Experimental parameters for demonstrated entanglement between macroscopic objects.

Target	Temperature (K)	Coherence time	Distance	Reference
Nd^3+^ Y_2_SiO_5_ crystal	3	7 ns	1.3 cm	5
10^12^ Caesium atoms	300	0.5 ms	few cm	2
10^5^ Caesium atoms	<1	1 *μ*s	2.8 m	3
Diamond crystal	300	7 ps	15 cm	4
^57^FeBO_3_ crystal	300	≲141 ns	~10 cm	this work

The case of the ^57^FeBO_3_ crystal is under theoretical investigation in this work.
